# Mode Split Equilibrium Microsimulation Approach for Early-Stage On-Demand Shared Automated Mobility

**DOI:** 10.3390/s22208020

**Published:** 2022-10-20

**Authors:** Lei Zhu, Jinghui Wang, Yuqiu Yuan, Wei Wu

**Affiliations:** 1 Department of Systems Engineering and Engineering Management, University of North Carolina at Charlotte, Charlotte, NC 28223, USA; 2 Department of Transportation Engineering, Shanghai Jiao Tong University (SJTU), Shanghai 200240, China; 3 School of Traffic & Transportation Engineering, Changsha University of Science and Technology, Changsha 410114, China

**Keywords:** simulation, automated mobility district, mode choice, shared mobility, equilibrium

## Abstract

The initial hype around Automated Vehicle (AV) technologies has subsided, and it is now being realized that near-term deployment of AV technologies will be in the form of low-speed shared automated shuttles in geofenced districts with a high density of trip demand. A concept labeled ‘Automated Mobility Districts’ (AMD) has been coined to define such deployments. A modeling and simulation toolkit that can act as a decision support tool for early-stage AMD deployments is desired for answering the questions such as (i) for a series of given conditions, such as the amount of travel demand and automated shuttle fleet configuration, what is the expected mode split for shared automated vehicle (SAV) services? (ii) for that mode share of SAVs, what level-of-service and network performance can be anticipated? To answer these research questions, an innovative and integrated framework of multi-mode choice and microscopic traffic simulation model is presented to obtain the equilibrium of mode split for various modes in AMDs, based on real-time traffic simulation data. The proposed framework was tested using travel demand and road network data from Greenville, South Carolina, considering a car, walk, and two SAV on-demand ridesharing modes in a proposed AMD. Results from the study demonstrated the efficacy of the proposed framework for solving the mode split equilibrium in an AMD. In addition, sensitivity analyses were conducted to understand the impact of factors such as waiting times and fleet resources on mode share equilibrium for SAVs.

## 1. Introduction

Automated vehicle (AV) technology is touted as the next big revolution in the transportation sector. While there is truth to that claim, widespread deployment of AV technologies is still far away [1]. In the short term, shared automated vehicles (SAV) [2] are being deployed in high-density geofenced neighborhoods (e.g., airports, university campuses, and downtown or uptown districts). Such deployments are labeled as automated mobility districts (AMDs), defined as district-scaled deployments of shared and automated mobility services to realize the full benefits of SAV technology in the near term [3,4,5]. AMDs have the dual advantage of serving as a cost-effective solution to short- and medium-distance trips and providing a firsthand experience of SAV technology to the public, eliminating the need to privately own an SAV. In AMDs, traditional public transit services are discouraged, and SAV transit services will be developed. AMD deployments are gaining traction in the United States [6]. In New York City (NYC), a fleet of six self-driving cars served passengers on the Navy Yards’ private roads through a loop shuttle service [7]. Columbus, Ohio, has launched two all-electric, autonomous shuttles that cruise starting and ending at the Linden Transit Center [8]. The above-listed early-stage deployments of AV and case studies indicate that a small-size fleet (2 to 6 vehicles) is usually applied and serves relatively small and confined areas. As cities move towards “shared mobility,” it becomes critical to have tools capable of accurately quantifying the impacts of such shared mobility services. 

Despite considerable research efforts in shared and automated mobility services from different perspectives, e.g., congestion releasing [9], mobility and energy benefits [10], travel efficiency [11], and so on, most of the existing studies focus on optimizing shared fleet operation through either mathematic approaches or simulation and heuristic approaches. Different mathematical programming models have been proposed with different focuses, such as dynamic route generation [12], traveler waiting for time minimization [13], service time window limitation [14], and electric vehicle charging efficiency [15]. Aside from coming up with solutions, simulations can evaluate the proposed algorithm under different scenarios [16] and quantify the benefits [17]. Microscopic simulation tools model detailed movements and locations of vehicles and people and would be more suitable to capture the nuances associated with modeling dynamic shared automated mobility systems. For example, Ruch et al. developed an open-source simulation testbed for autonomous mobility-on-demand systems–AMoDeus [18], based on the agent- and activity-based transport simulation framework MATSim [19] and the Dynamic Vehicle Routing Package [20]. However, most state-of-the-art microscopic models focus on private cars or transit buses and have an inadequate representation of SAVs. Also, most models assume optimized fleet size for a given demand under service performance constraints. Few studies [21,22], if any, have focused on quantifying rideshare service characteristics when the supply resources (such as vehicle fleets) are limited. Besides, those models assume that shared vehicles are standalone without capturing the interactions between shared mobility services and network traffic. Conversely, network performance and levels of service can affect the choice of shared mobility service and operations of shared vehicles. Almost no academic work reviewed in this field has been converted into an open-source, easy-to-use decision support toolkit that can inform real-world AMD deployments regarding the performance of SAVs services. This necessitates a modeling and simulation toolkit that can accurately assess the mobility and energy benefits of deploying SAVs in the real world. 

On the other hand, a few researchers have briefly studied mode choice models with on-demand ridesharing modes. Various kinds of factors and models were studied to explore user preference between different choices, such as SAV modal split [23], regular vehicles versus PAVs and SAVs [24], ride sharing versus privacy [25] as well as preference heterogeneity [26]. For example, a traveler chooses a model with SAV modes depending on the travel distance, trip duration, and waiting time based on a multinomial logit (MNL) mode choice model described the impact of SAV modes [27]. The choice of a shared mode is affected by an individual’s socio-demographic characteristics, such as income level, education, as well as service characteristics of the mode, and network performance. Nevertheless, assuming the homogenous socio-demographic characteristics of all individuals, the choice of shared modes depends heavily on network traffic conditions and the performance of the mobility services. In turn, the mode choices of individuals will update network traffic conditions and change the level of service of the shared modes, thus affecting the next round of choice. Such an iterative procedure keeps improving mode choice results towards equilibrium for shared mobility modeling. 

In practice, the SAV fleets in early-stage AMD deployments will be limited owing to budget constraints and are likely to start with a few vehicles and then gradually increase the fleet size as necessary [28]. Therefore, understanding the impact of the level of service (LOS) parameters (wait time, travel time, etc.) of new mobility options with SAVs will greatly help city transportation planners and authorities make investment decisions in deploying SAV fleets. Also, although there are many studies focusing on SAVs from various perspectives, such as finding an optimal fleet route with mathematical programming [12,13] or simulation approaches [16,17], considering the service performance constraints [21,22] as well as studying mode choice models with on-demand ride sharing modes [23,24], there are some points that have been ignored. The research gap is threefold: few research studies considered different modes existing simultaneously in the SAV routing problem; few studies have focused on quantifying rideshare service characteristics when the supply resources are limited; and the interaction between shared mobility services and network traffic has been mostly ignored. Therefore, the research question is: Given the specified travel demand, a limited number of SAVs, and specific mobility costs (such as the unit cost of travel distance or travel time), how can a mode choice solution be determined for all modes, including SAV modes, while achieving a specific objective (i.e., minimizing passenger waiting time) and maintaining the required network LOS?

This study addresses the proposed research gaps and develops a microsimulation-based multi-modal equilibrium approach for on-demand shared and automated mobility services with limited fleet resources. The model introduces an iterative procedure in which mode choice is continually updated to ultimately reach an equilibrium solution. Along with the mode choice equilibrium solution, the service performance of SAVs generated by the proposed toolkit can inform cities regarding the benefits of emerging mobility modes. Four travel modes, namely: (i) on-demand door-to-door ridesharing, (ii) on-demand fixed-route ridesharing, (iii) private car, and (iv) walking, were considered for the modeling exercise. The Simulation of Urban Mobility (SUMO) package [29] was used for the network simulation, given its unique capability in characterizing ridesharing, vehicle connectivity, and automation features. Therefore, the primary contributions of this study are:

- Development of a sophisticated and extensible open-source microscopic simulation platform for describing multiple on-demand ridesharing (door-to-door and fixed-route) services in AMDs considering regular traffic and automated shared mobility and their interactions. The developed tool will provide detailed simulation performance of new mobility options in the network and could be used for assessing on-demand ridesharing in terms of mobility and energy;
- Integration of mode choice and simulation model to reach an equilibrium solution where mode split and level of service of SAVs become stable for the early deployment of AMDs. As a result, the supply potential of new mobility modes can be evaluated. The valuable assessment results assist and support the transportation stakeholders and decision-makers in advancing emerging on-demand shared mobility.

The rest of the paper is organized as follows: the Section 2 section describes the mode choice, simulation framework, and detailed algorithm. The Section 3 tests the proposed framework in a real-world road network in Greenville, South Carolina, shows the simulation results, and conducts the sensitivity analysis. Finally, the Section 4 summarizes the paper and proposes some topics for future study.

## 2. Methodology

### 2.1. Mode Choice and Simulation Iterative Framework

**Figure 1:**
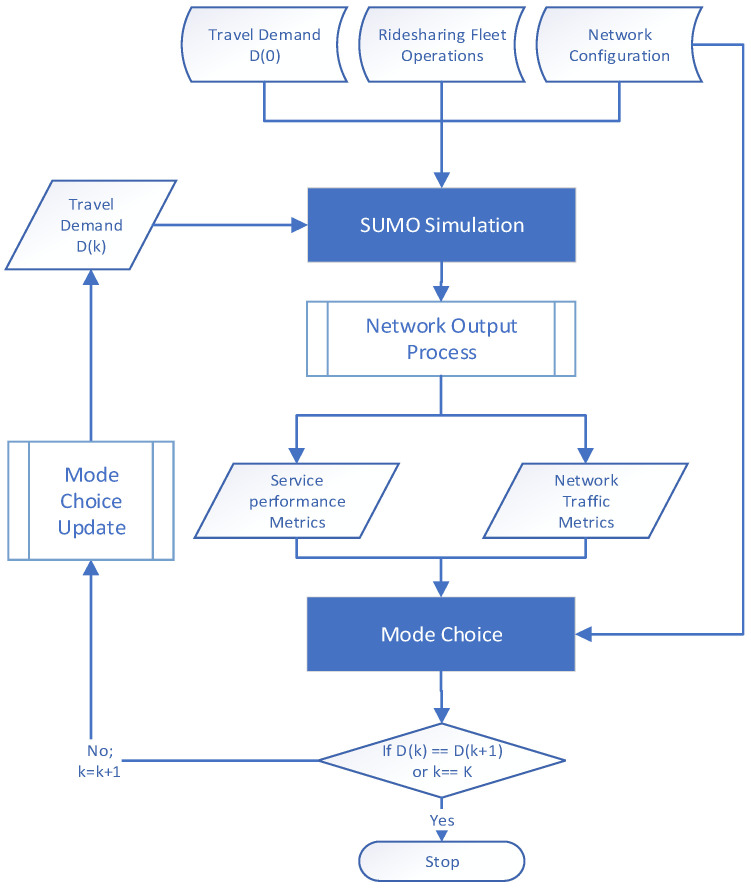
Framework of the simulation-based shared mobility model.

A trip in an AMD is expected to have the following four trip elements (at a minimum): origin, destination, departure time, and travel mode. The modal spectrum considered in this study comprises (1) regular car (CAR), (2) pedestrian (WAK), (3) on-demand door-to-door ridesharing (DTD), and (4) on-demand fixed-route ridesharing (FXR) modes. The DTD and FXR modes are the two shared mobility services, both of which are served by SAVs. The DTD mode is able to conduct pickups and drop-offs anywhere in the network, and the SAVs will be driving within the entire road network. In contrast, the FXR mode SAV runs on designated routes with fixed SAV stops. FXR SAVs are running on the existing roads along a fixed route, which do not need guideways. The passengers must move to the fixed SAV stops/depots to be picked up and dropped off. The designated route is considered as a narrow and small-scale “network”. The SAV routing on such designated routes or “small-scale networks” leads to fixed routes with identical travel distances for specific OD pairs. The proposed framework illustrated in Figure 1 resolves the mode choice problem iteratively to achieve equilibrium and a feasible solution.

The research problem is to find an equilibrium solution for mode choice $D^{*}$ through an iterative procedure between two major modules: the SUMO simulation module and the mode choice module. Initially, the SUMO simulation module takes the initial mode choice $D\left(0\right)$ at iteration 0, along with network and SAV configurations to simulate all trips and thus produces service performance metric δ, which could be any metrics of the shared service performance, such as passenger waiting time, trip detour factor, travel distance, travel time. In this study, passenger waiting time is used for describing the shared service performance, which will be introduced in the network outputs section. If more trips select SAV modes, given limited and specific SAV supply, the passenger waiting time will be increased. In comparison, the long waiting time decreases the probability of the SAV mode choice in the next iteration. In that case, the equilibrium of mode choice will ultimately be reached. A network output process follows the SUMO simulation module to process the simulation outcomes and generates two types of performance metrics- service performance metrics and network traffic metrics. Those two metrics will be fed to the mode choice module to update mode shares to $D\left(k\right)$ at iteration k, based on δ and a pre-defined passenger waiting time threshold $\Delta_{m},\;m\in \left\{DTD,\;FXR\right\}$ for shared modes (non-shared modes don’t have such thresholds). The passenger waiting time threshold $\Delta_{m}$ represents the minimum LOS required from the ridesharing modes. The iterative procedure is terminated either when the mode choice result is consistent between two consecutive iterations or when the iteration indicator k reaches its limit K. The mode split result is said to reach equilibrium or stable if the results of two consecutive iterations k−1, and k  converge, as defined by the convergence factor $r_{k}$ shown in Equation (1).

(1) $$r_{k}=\frac{\left|D\left(k-1\right)\cap D\left(k\right)\right|}{\left|D\left(k-1\right)\right|}$$

where $\left|D\left(k-1\right)\right|$ denotes the total number of trips (of all modes) in iteration k−1, and $\left|D\left(k-1\right)\cap D\left(k\right)\right|$ denotes the number of trips across iterations k−1,  and  k with the same mode choices. For a user-provided convergence threshold σ, if the condition $r_{k}>\sigma$ is satisfied (say σ=0.995), the iterative procedure is considered to converge; otherwise, the procedure continues to update the mode choice D for the next iteration or until it reaches the maximum iteration limit K. When the iterative procedure converges, the result indicates an equilibrium of mode choice satisfying LOS requirements for the SAV modes. That is defined as a feasible solution of the framework to find a mode choice solution for all OD pairs, which makes the solution not change by iterations (stable) and satisfies the objective and constraints (e.g., minimizing passenger waiting time). 

### 2.2. SUMO Simulation

The simulation platform adopted for the development of the AMD toolkit is SUMO–Simulation of Urban Mobility, an open-source, microscopic multi-modal traffic simulation package. The Traffic Control Interface (TraCI) API [30] is one such extension that enables SUMO simulation to interact with external applications to realize online interactions, such as accessing and changing simulation values. The simulation platform leverages TraCI APIs to implement two on-demand ridesharing services (DTD and FXR). For regular car mode, a time-dependent shortest path (TDSP) considering simulation traffic conditions is applied, while the distance-based shortest path on the walkable network is used for pedestrian routing. 

#### 2.2.1. Shared Vehicle Dispatching Operations 

Ride sharing modes DTD and FXR share a similar dispatching logic, which consists of the steps below: 

System Status Collection: This step collects the information regarding available vehicles and potential passengers in the network at every time interval t∈T, such as *t* = 300 s, where T is a simulation horizon. *Potential passengers* are the people who choose an SAV mode and request a ride during the system status check period. It is assumed that only one passenger makes each ride request. *Available SAVs* are vehicles with spare seats and ready-to-serve passengers (no uncompleted ride tasks). 

Ride Matching and Vehicle Routing: *An incremental spatio-temporal matching* is proposed to match each passenger to a vehicle. First, potential passengers are sorted in ascending order based on their departure times. Then, the first passenger in the sorted list is assigned priority and matched with the nearest available vehicle. The distance between the requests and the available vehicles is calculated by a time-dependent shortest path (TDSP) at the request time, which considers dynamic network traffic patterns in route choice and has been widely used for simulation studies [31,32]. This procedure continues until all passengers are matched with available vehicles or all available SAVs find their matched passengers based on a time-distance search. A vehicle is allowed to serve multiple passengers simultaneously. The algorithm (Algorithm 1) outputs the mapping dictionary M for vehicle routing. The details of the algorithm are illustrated below.

 **Algorithm 1: Temporal-Spatial Incremental Matching Algorithm**
 Vehicle capacity dictionary: $C=\left\{v:seatCapacity,\ldots \right\}$
 Passenger-vehicle mapping dictionary: $M=\left\{v:\left[r_{1},\;r_{2},\ldots \right],\ldots \right\}$

 ***For*** 
t∈T
*:*
 1. Data collection
    a. Potential passengers, $R_{t}=\left\{r_{1},r_{2},\ldots ,r_{m}\right\}$
    b. Available vehicles, $V_{t}=\left\{v_{1},v_{2},\ldots ,v_{n}\right\}$ 2. Ride Matching # sort potential passengers by departure time, in ascending order
      $\hat{R_{t}}=sortedDT\left(R_{t}\right)$
    ***for***
r
***in***
$\hat{R_{t}}$*:*    # sorted $V_{t}$ by distance from each v to r, ascendingly        ${\hat{V}}_{t}\left(r\right)=sortedDist\left(V_{t},\;r\right)$    ***for***
v
***in***
${\hat{V}}_{t}\left(r\right)$*:*        ***if***
$\left|M\left[v\right]\right|<C\left[v\right]$*:*                $M\left[v\right]=M\left[v\right]\cup \left\{r\right\}$                ***break***            ***else:***                ***continue*** ***output:***
M


Given the matching dictionary M, the vehicle routing generates a route plan with a pickup and drop-off service sequence and a time-dependent shortest paths (TDSP) route chain. A First-In-First-Out (FIFO) logic is applied to determine the pickup and drop-off sequence for each vehicle in order to simplify the problem. All passengers were picked up first and then dropped off in the order of the passengers in the mapping dictionary M. To travel between locations, vehicles follow TDSPs, which account for real-time traffic congestion on the network. For instance, if $M=\left\{v:\left[r_{1},\;r_{2}\right]\right\}$, the pickup and drop-off order of vehicle v is $p\left(r_{1}\right),\;p\left(r_{2}\right),d\left(r_{1}\right),\;d\left(r_{2}\right)$, where $p\left(\cdot \right)$ and $d\left(\cdot \right)$ denote the vehicle pickup and drop-off actions, respectively. With that, an SAV takes TDSPs to link the pickup and drop-off locations.

It is worth noting that to simplify the problem and increase the simulation speed, the relatively simple logic of ride-matching and vehicle routing is introduced, as mentioned above. The ride-matching and vehicle routing problems have been intensively studied recently. For example, some research activities focus on minimizing total travel distance or travel time, and sophisticated vehicle routing algorithms are always needed in this area. However, developing vehicle dispatching models or algorithms in this study is out of scope. 

Redistribution: The redistribution procedure re-locates unoccupied vehicles to a new place in anticipation of future trip demand. Vehicle relocation aims at reducing passengers’ waiting time and improving the service efficiency of SAVs. For the simplified case study analysis presented in this paper, each SAV is made to simply stop at the location where the latest passenger was dropped off. 

#### 2.2.2. Passenger Actions 

**Figure 2:**
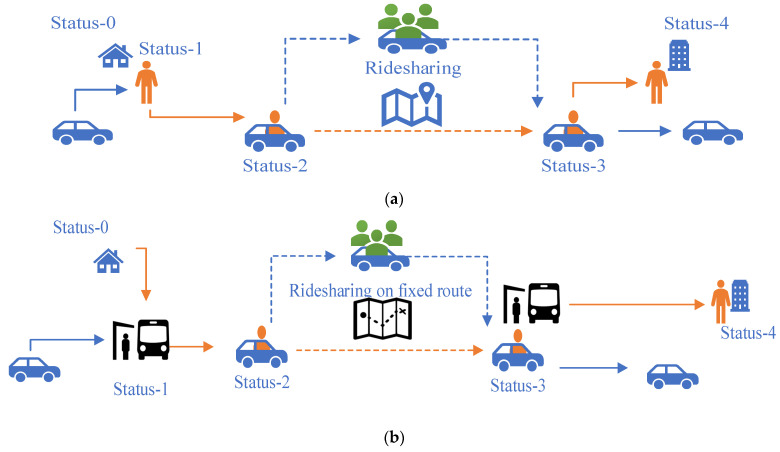
Passenger behavior description for: (**a**) on-demand door-to-door ridesharing service; and (**b**) on-demand fixed-route ridesharing service.

During a trip, each ridesharing passenger in the simulation environment has five action states: 0—initialization; 1—arrive at the pickup location and wait; 2—get onboard; 3—arrive at the drop-off location and alight; 4—arrive at the destination and stop. As noted before, the two ridesharing modes share the same vehicle dispatching logic but differ slightly in operation. Figure 2 provides vivid descriptions of passenger actions in each state and compares the two ridesharing modes. The orange lines represent the passengers’ behavior. The blue lines indicate the vehicle operations.

#### 2.2.3. Network Outputs

The network outputs from the simulation module include (1) service performance metrics and (2) network traffic conditions. The network traffic condition is depicted by link-level statistics consisting of average speed (mph) and travel time (s) in a specific time interval, which supports the TDSP calculation. The service performance metrics are computed based on SUMO simulation results, such as vehicle trajectory and passenger loading. The service performance metrics for the two ridesharing modes and car mode consist of the following:

- Vehicle miles traveled (VMT) in miles for DTD, FXR, and CAR modes;
- Vehicle energy consumption (VEC) in gallons for DTD, FXR and CAR modes. In this study, to simplify the problem, VEC is defined as the fuel consumption by all vehicles. The characteristics of all vehicles in the simulation are set to match those of a standard, midsize sedan such as the Toyota Camry 2016. The VEC is calculated using the detailed second-by-second driving cycles from SUMO along with the FASTSim simulation model. More details of the vehicle powertrain and model can be found in [5];
- Vehicle travel time (VTT) in seconds for DTD, FXR, and CAR modes;
- Vehicle deadheading distance (VDH) in miles for DTD and FXR modes;
- Vehicle loading rate (VLR) for DTD and FXR mode. VLR defines the number of passengers onboard weighted by the vehicle distance traveled for all SAVs. VLR indicates a vehicle’s efficiency in transporting more people per mile of travel;
- Vehicle detour factor (VDF) for DTD and FXR modes. VDF is calculated as the trip distance of ridesharing modes divided by the trip distance of the regular car mode of TDSP. Therefore, an efficient rideshare mode is expected to have a lower VDF;
- Passenger waiting time (PWT) in seconds for DTD and FXR modes. PWT is defined as the time difference between the request time and pickup time;
- Passenger walking time (WKT) in seconds for FXR mode.

### 2.3. Mode Choice Model

Adopting the multinomial logit (MNL) mode choice model from Liu [27], a mode choice model considering SAV modes together with car and walk modes is proposed in this study (Equations (2)–(6)). Equation (2) is used to calculate the choice probability of choosing mode m.

(2) $$P\left(m\right)=\frac{\exp\left(V_{m}\right)}{\sum_{m}\exp\left(V_{m}\right)},\;\forall m\in \left\{\mathrm{CAR},\;\mathrm{WAK},\;\mathrm{DTD},\;\mathrm{FXR}\right\}$$

The utility functions of the four travel modes considered in this study are illustrated below. Walk mode is considered as the base alternative in the mode set. Utility functions of the two ridesharing modes are formulated considering a pre-defined waiting time threshold. The utility function of each mode is composed of the following components: constant cost or fare (const), distance cost (dist), in-vehicle travel time cost (ivtt) and two out-of-vehicle travel time cost (waiting time for SAV ($ovtt_{wait}$) and walking time to departure SAV stops ($ovtt_{walk}$)). The units of distance and travel time are “mile” and “hour”.

(3) $$V_{\mathrm{CAR}}=-15.3-0.6*dist^{\mathrm{CAR}}-17.67*ivtt^{\mathrm{CAR}}\;$$

(4) $$V_{\mathrm{WAK}}=-1.2*dist^{\mathrm{WAK}}-35.34*ovtt_{walk}^{\mathrm{WAK}}$$

(5) $$V_{\mathrm{DTD}}=\left\{\begin{array}{cc} -0.83 & -0.34*dist^{\mathrm{DTD}}-8.84*ivtt^{\mathrm{DTD}}-35.34*ovtt_{wait}^{\mathrm{DTD}},\;if\;ovtt_{wait}^{\mathrm{DTD}}\leq \Delta_{\mathrm{DTD}}\\ -\infty , & if\;ovtt_{wait}^{\mathrm{DTD}}>\Delta_{\mathrm{DTD}}\; \end{array}\right.$$

(6) $$V_{\mathrm{FXR}}=\left\{\begin{array}{cc} -2-8.84*ivtt^{\mathrm{FXR}}-35.34*\left(ovtt_{walk}^{\mathrm{FXR}}+ovtt_{wait}^{\mathrm{FXR}}\right), & if\;ovtt_{wait}^{\mathrm{FXR}}\leq \Delta_{\mathrm{FXR}}\\ -\infty , & if\;ovtt_{wait}^{\mathrm{FXR}}>\Delta_{\mathrm{FXR}} \end{array}\right.$$

From the constant cost (const) perspective, the fixed cost of car mode, including vehicle maintenance cost, parking cost, and the congestion charge, is set as $15.3 per trip following [33], which also proposes a $0.83 constant cost for “Uber-like” DTD mode. FXR mode has a constant cost of $2 per trip, following transit fare assumptions used by Liu et al. [27]. We assume the constant cost of FXR mode is the same as the transit flat fare, although the FXD mode has an on-demand feature. Walk mode doesn’t have any constant cost in the model. For the distance cost (dist) component, the distance-based unit cost of regular car mode is $0.6 per mile [34], and in walk mode, it is set as $1.2 per mile following [33]. It is worth noting that for relatively short trips in AMDs, the value of the distance term is minimal than that of other costs, such as travel time term in walk mode. Leaving the distance cost in the walk mode function aims to keep the formulation consistent. For DTD mode, the cost is set as $0.34 per mile [33]. Since transit mode observes flat fares, the transit-like FXR mode is assumed that it doesn’t have a distance-based cost term [27]. The cost of taking transit is somehow fixed based on the lines and riding services, not based on the trip distance [27]. The unit cost of in-vehicle travel time (ivtt) of CAR mode is defined as $17.67 per hour [35], while those for DTD and FXR are assumed to be 50% of that in the CAR mode, $8.84 per hour, as passengers choosing SAV mode don’t need to drive and are able to perform other activities such as working or resting. There is no ivtt term for walk mode. From the out-of-vehicle travel time cost (ovtt) perspective, no ovtt cost is applied for CAR mode. Contrarily for walk mode, only cost of $ovtt_{walk}$ is considered. For DTD mode, wait time ($ovtt_{wait}$) is considered rather than walking time. In the case of FXR mode, both $ovtt_{walk}$ and $ovtt_{wait}$ are included. The unit costs of out-of-vehicle travel time are uniformly set as $35.34, which is two times that of ivtt [36].

For car mode, TDSP is computed based on existing traffic conditions, which provides the dist and ivtt values of the mode. Similarly, for the walk mode, minimum distance paths based on network topology inform the dist variable for the trip. The speed of walk mode is set as 5 kmph to convert dist to $ovtt_{walk}$ in simulation. With the constant costs, the car and walk modes’ utility values can be computed. 

For DTD mode, the travel distance (dist) and travel time (ivtt and $ovtt_{wait}$) of a trip are obtained from the simulation. At the beginning of the simulation, dist, ivtt and $ovtt_{wait}$ are constant for all trips. If a trip is assigned to DTD mode, the dist,ivtt and $ovtt_{wait}$ produced by the simulation will replace those of the trip for computing a new mode choice utility value. Inputs required for the utility function of FXR mode are calculated differently than DTD mode. First, two SAV stops closest to a trip’s origin and destination, respectively, are identified. The minimum travel time between the two stops along the fixed SAV route is estimated and used as the ivtt for the trip. Further, walking distances from the origin and destination to their corresponding nearest SAV stops are computed using the logic described for $ovtt_{walk}$. Finally, the waiting time of the FXR mode $ovtt_{wait}$ is estimated similarly to that of the DTD mode. 

Once all inputs are available, the utility and the probability of each mode are computed using Equations (2)–(6). The mode with the maximum probability is assigned to the trip. To maintain an expected level of service for shared modes, an upper bound is set on passenger waiting time, beyond which the travelers do not choose shared modes. 

#### Mode Choice Update

In order to avoid mis-convergence and slow convergence, it is necessary to balance the mode choice probabilities of two consecutive iterations while computing new choice probability values for determining the mode shares of the trip. Therefore, a set of probability update methods is used, as shown in Equation (7).

(7) $$P{\left(m\right)}_{k^{\prime }}=\left\{\begin{array}{c} P{\left(m\right)}_{k-1}+\frac{1}{k}*\left(P{\left(m\right)}_{k}-P{\left(m\right)}_{k-1}\right),\;\;\;\;if\;m=CAR\\ P{\left(m\right)}_{k-1}+\frac{\left|ovtt_{wait}^{m}-\Delta_{m}\right|}{\max\left(ovtt_{wait}^{m},\;\Delta_{m}\right)}*\left(P{\left(m\right)}_{k}-P{\left(m\right)}_{k-1}\right),\;if\;m=DTD\;or\;FXR\\ \max\left(0,\;1-P{\left(CAR\right)}_{k^{\prime }}-P{\left(DTD\right)}_{k^{\prime }}-P{\left(FXR\right)}_{k^{\prime }}\right),\;\;\;\;if\;m=WAK \end{array}\right.$$

where k  indicates the iteration number, and $P{\left(m\right)}_{k^{\prime }}$ is the updated probability at iteration k. The successive averages (MSA) method updates choice probability for car mode. For DTD and FXR modes, a waiting time weighted (WTW) MSA method is introduced by applying the term $\frac{\left|ovtt_{wait}^{m}-\Delta_{m}\right|}{\max\left(ovtt_{wait}^{m},\;\Delta_{m}\right)}$, which represents a ratio ranging from 0 to 1 and describes the “distance” or “discrepancy” of waiting time to its targeting threshold. When the waiting time is close to the threshold, the term approaches 0; otherwise, it closes to 1. From Equations (5) and (6), if the waiting time $ovtt_{wait}^{m}\;$ is greater than the threshold $\Delta_{m}$, the utility of the mode m reaches negative infinity, resulting in a close-to-zero choice probability $P{\left(m\right)}_{k}$ for that mode. This generates $P{\left(m\right)}_{k^{\prime }}=\left(1-\frac{\left|ovtt_{wait}^{m}-\Delta_{m}\right|}{\max\left(ovtt_{wait}^{m},\;\Delta_{m}\right)}\right)*P{\left(m\right)}_{k-1}$ according to Equation (7), implying that if the waiting time resulted from the choice at the last iteration is higher than the threshold, it may result in a lower probability of choosing the mode at the current iteration than the previous iteration; the larger “distance” of the waiting time to the threshold, the lower the probability. Therefore, in the case where $ovtt_{wait}^{m}>\Delta_{m}$, the weighted constraint $\frac{\left|ovtt_{wait}^{m}-\Delta_{m}\right|}{\max\left(ovtt_{wait}^{m},\;\Delta_{m}\right)}$ is essentially a penalty of waiting time beyond the threshold, which would discourage the use of the shared mode. If the waiting time is less than the threshold, it is favorable to hold the waiting time constraint. In such cases, the $P{\left(m\right)}_{k^{\prime }}$ will be a balanced value between $P{\left(m\right)}_{k}$ and $P{\left(m\right)}_{k-1}$. 

For walk mode, its new probability is estimated either as 0 or the remainder from all other modes (i.e., walk mode is considered as the base alternative) to ensure that the probability summation of all modes is 1. 

## 3. Case Study

**Figure 3:**
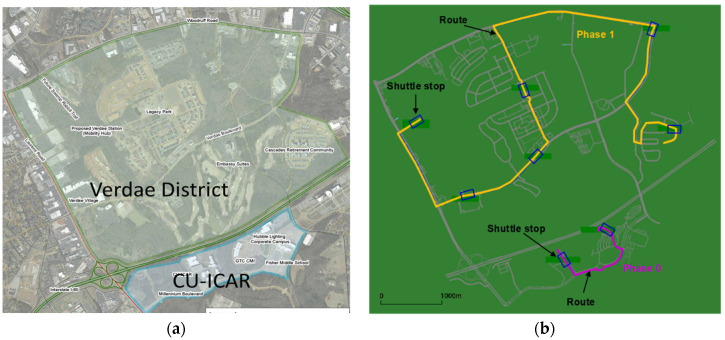
A-Taxi test field phase 0 (CU-ICAR) and phase 1 (Verdae District) at Greenville, SC: (**a**) satellite image; and (**b**) AMD simulation network and fixed-route configuration.

The proposed modeling framework was tested in the context of a proposed automated taxi (A-taxi) deployment in Greenville, South Carolina. The Greenville A-Taxi (or AMD) deployment consists of two phases described below and depicted in Figure 3a.

### 3.1. Simulation Network Preparation

Data for the Greenville deployment was extracted from the Greenville regional travel demand model maintained by Greenville-Pickens Area Transportation Study (GPATS). The dataset is frequently maintained and equivalent to the calibrated data for simulation. First, the road network was imported into SUMO, followed by network editing and pre-processing [37,38]. The editing process consisted of removing disconnected road segments, classifying road segments by functional class, checking the number of lanes on each segment, and checking traffic signal locations. The default signal phasing feature available in SUMO is used to define the timing for all signals in the simulation. The simulation includes four modes (CAR, WAK, DTD, FXR). DTD fleet comprises **four** SAVs that can operate anywhere in the road network. FXR mode comprises a fleet of **six** SAVs, two of which operate on a fixed route in Phase 0 (see purple lines in Figure 3b), and the remaining four SAVs operate on the fixed route in Phase 1 (see yellow lines in Figure 3b). Due to the light traffic of Phase 0, to simplify the simulation, two SAVs in Phase 0 will be ignored, and only four SAVs in Phase 1 will be considered as the FXR mode fleet in the simulation. Therefore, a total of eight SAVs will be running in the study. Each SAV has a seating capacity of four passengers. The routes and shuttle stops are illustrated in Figure 3b.

The AMD deployment region is within eight TAZs (about two square miles). While the travel data of Greenville comprises four tables (AM Peak (6–9 a.m.); Mid-day (9 a.m.–4 p.m.); PM Peak (4–7 p.m.); and Night (7 p.m.–6 a.m.)), the case study uses only AM peak data (comprising of 308 trips) for testing. The trip demand is spread across the entire AM peak period using a hypothetical normal distribution. SUMO simulation is calibrated by using a “calibrator” object to adapt dynamic traffic flows, speeds, and vehicle parameters based on limited real-world link speed measurements (from a public web resource of the area). The calibrated simulation indicates that the travel time results match the real-world observations for such a small area with light traffic. For this Greenville study, one simulation for each iteration will take about 3 min in an ordinary laptop computer with an Intel i7-4600U CPU, 16G RAM, and a Windows 10 Enterprise 64-bit operating system. A python script of mode choice utility functions is implemented. The mode choice results will be fed into the SUMO simulation to get service performance metrics and network traffic metrics, which will be used for the mode choice module. The integration and iteration are also implemented as a python script running online. 

### 3.2. Simulation Results

**Figure 4:**
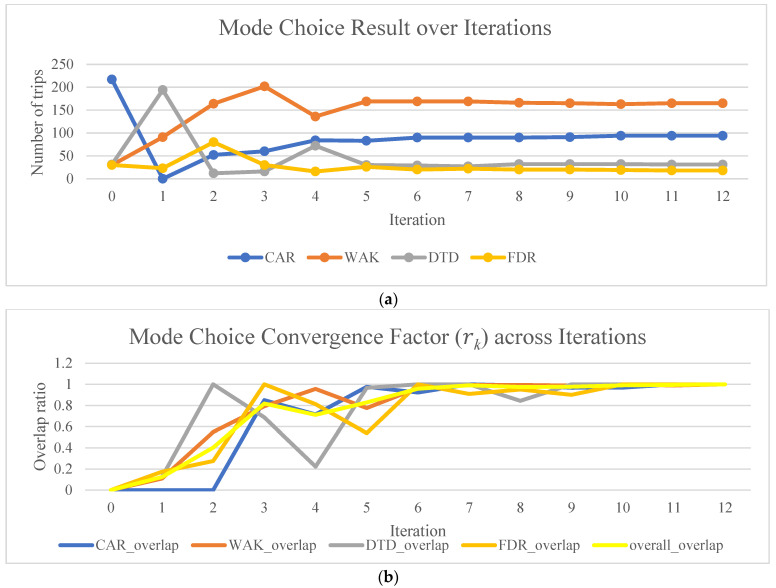
Mode choice results over iterations (**a**) trip frequencies, (**b**) overlapping rates.

The simulation (iteration 0) was initialized with a random seed that generated 217, 30, 31, and 30 trips for all four modes. A large waiting time threshold (i.e., 500 min) was set for the SAV modes in order to mitigate any impacts of waiting time constraints on the convergence of mode choice results. Link-based performance metrics, including average speed (m/s), were updated every 5 min for computing TDSP. SAV performance metrics are computed using the vehicle trace data, vehicle stop, and resharing log data from the simulation. The mode choice results for consecutive iterations are presented in Figure 4. Mode shares fluctuate drastically in the first few iterations before they start converging around iteration 10, resulting in mode shares of 94, 165, 31, and 18 trips for CAR, WAK, DTD, and FXR modes, respectively. 

A noteworthy observation here is that mode shares between the initial setting (217, 30, 31, 30) and the converged result (94, 165, 31, 18) in terms of the number of trips, travelers have a significant shift from CAR to WAK mode while fewer shifts are observed for DTD and FXR modes. These shifts in demand may be caused by the mobility costs in the mode choice model and the interaction among the four modes. The increasing and converging trend of mode choice overlapping ratios in Figure 4b shows that for individual trips, the mode choices remain unchanged, which means the trip mode choice reaches an equilibrium. It can be observed that the proposed iterative framework was able to provide an equilibrium mode choice solution within a reasonable number of iterations. 

Table 1 presents the simulation performance metrics of all modes. It is worth noting that the walk mode has no direct impact on traffic in the simulation experiment. The average trip distance of walk mode is only 0.79 miles, which is significantly lower than other modes, while from a travel time perspective, walk mode has a higher average trip travel time. Although FXR mode has six SAVs available, only four SAVs (the ones in phase 1) were used in the simulation depending on the trip requests because the number of trip requests in phase 0 was very low. Comparing the shared modes, DTD has a higher VDH/VMT ratio which is very close to the VDH/VMT ratio for TNC vehicles such as Uber and Lyft [39]. This is consistent with expectations since DTD mode can pick up and drop off passengers anywhere in the network, leading to detours with empty vehicles. FXR mode, however, travels on a fixed route and is not allowed any detours. Therefore, the average vehicle passenger loading (AVG. VLR) is higher for FXR mode since travelers may need to cluster at SAV stops, which could increase vehicle occupancy. The overall VMT and VTT values are higher for car mode than other modes because there are more cars in the network than SAVs. 

At the trip level, DTD mode has a lower average VMT, VTT, and VDF than FXR mode, which may be attributed to the routing flexibility of DTD mode. On the other hand, CAR mode beats both shared modes in average VMT, VTT, and VDF, as private cars don’t need to detour for ridesharing. Also, the VEC is proportional to VMT. As for the level of service, the average passenger waiting time (AVG. PWT) for both SAV modes was close to 300 s. However, FXR mode has an extra 990 s of walk time per trip, making it slightly unattractive compared to DTD mode. 

Simulation results demonstrate the benefits of the use of the SAV mode. A total of 49 trips are served by 8 SAVs, meaning each SAV can replace 6 regular cars to serve trip requests. This benefit, however, comes with a cost in that passengers must wait longer (as evidenced by PWT, WKT factors), and vehicles have to travel longer (as evidenced by VDF factor) in the network, meaning a higher amount of energy consumed. While the electrification of SAV fleets can mitigate some of these adverse effects, sophisticated fleet dispatching strategies, along with an increased demand for these services, will provide a better solution for this problem. 

### 3.3. Sensitivity Analysis and Discussion

#### 3.3.1. Waiting Time Constraint

**Figure 5:**
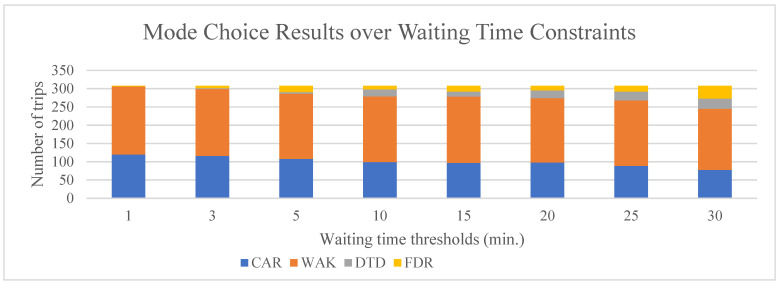
Mode choice results over waiting time constraints.

To understand the sensitivity of mode choice to SAV waiting times, a set of waiting time thresholds (from 1 min to 30 min) were tested, with all other simulation settings remaining the same. The equilibrium results are presented in Figure 5. As expected, mode shares for DTD and FXR increase gradually with the increase in the waiting time thresholds, which means a loose waiting time constraint. However, according to the diagram, the mode share seems not to change much at about 20 min waiting time threshold. 

An interesting observation here is that the share of the SAV modes shifts directly from the CAR mode, with the walk mode almost unchanged. This indicates that, as waiting time thresholds change, the car’s generalized cost (GC) and SAV modes become comparable, whereas GC for the walk mode is far lower than any other modes, making the mode shift from walk difficult.

#### 3.3.2. Fleet Size and Seating Capacity

Given the low travel demand for FXR mode in phase 0 of the Greenville AMD, the analysis ignored the FXRs in phase 0. Various scenarios were the varying fleet size and vehicle capacity from 1 to 4, with all other configurations remaining the same. The combinations of fleet size and vehicle capacity are represented as “(DTD fleet size − FXR fleet size) × (DTD vehicle capacity − FXR vehicle capacity)”.

**Figure 6:**
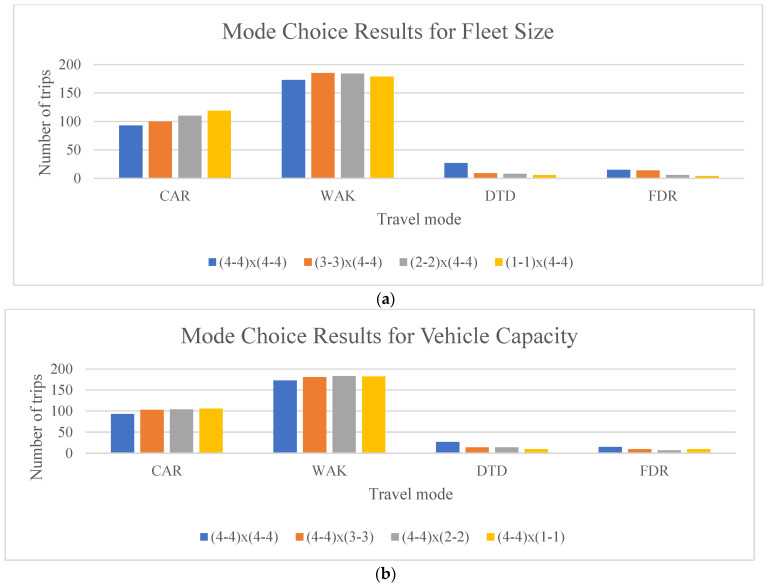
Mode choice results for different fleet sizes (**a**); and vehicle capacities (**b**).

Figure 6a shows the analysis results where fleet size varies from 4 to 1 while keeping the seating capacity constant as fleet size drops, SAV modes lose mode share to car mode. For DTD mode, the loss of mode share is high when the fleet size is reduced from 4 to 3 vehicles, while for FXR mode, the loss of mode share is more pronounced when the fleet size is reduced from 3 to 2 vehicles. Findings such as these will help decision-makers devise investment strategies to maximize the benefits while minimizing costs. Figure 6b shows the analysis results where the seating capacity is reduced from 4 to 1 while maintaining a constant fleet size for two SAV modes. A decrease in seat capacity has a more gradual mode share reduction effect than reducing fleet size. Trade-offs between fleet size and set capacity of SAVs will help right-size the fleet for a given deployment context.

## 4. Conclusions

This paper describes the AMD toolkit and proposes a modeling framework that connects network simulation and mode choice components of the toolkit to continuously update mode choice for various modes in early-stage AMDs through an iterative updating procedure based on network conditions from the previous iteration. The proposed framework was tested using real-world travel data from a proposed AMD deployment in Greenville, SC. Results from the case study prove that the framework could provide an equilibrium mode choice solution within a reasonable number of iterations. Further, the performance metrics for various modes obtained from the simulation results align with mode shares observed for respective modes. Next, the sensitivity of the mode share equilibrium procedure was tested using parameters such as Level-of-service (passenger waiting time), fleet size, and vehicle capacity. Results reveal that relaxing waiting time constraints increases the share of SAV trips and that the mode share of SAV drops with decreasing fleet size and seat capacity.

Future efforts will focus on disaggregating parking parameters in the utility equation to better understand trade-offs in mode choice between areas where parking is scarce (say downtown) versus where parking is freely available. Since the simulation tool is flexible and compatible, more public transit modes and SAV impact on transit demand will be modeled. Due to the lack of early-stage deployments of AMDs in the real world and ground real traffic data, how the proposed framework performs using large-scale demand and supply data and the comparison between the simulation against real data will be explored if the availability of real data and other conditions are satisfied. The real traffic data of the AMDs should be used for simulation model calibration. Although the proposed approach was tested on a small-scale network, the results indicate that it is promising to work on large networks with high demands. It is expected that more computational time and resources are needed to implement it and thus achieve the mode split equilibrium result. That means a high-power-computing (HPC) solution may need for large-scale applications. Finally, since the pilot study focused on a small and relatively isolated area, the SAV services were primarily designed to satisfy internal trips in the region. The SAV is expected to reduce traffic congestion within the area because one SAV could replace more single-passenger vehicles. Therefore, the area with SAV services may attract more trips from other areas. The SAV impact on the network performance of the surrounding areas should be explored in future research. 

## Figures and Tables

**Table 1:** Performance Metrics of Mode Choice Results.

*METRICS*	DTD	FXR	CAR	WAK *
**OVERALL MOBILITY PERFORMANCE**				
***# OF VEHICLES***	4	4	94	165
***TOTAL VMT (MILE)***	110	87	223	130
***TOTAL VDH (MILE)***	60	43	0	-
***VDH/ VMT***	0.55	0.49	0	-
***TOTAL VTT (SEC.)***	20,022	15,297	30,013	174,587
***AVG. VLR***	0.49	0.72	1.00	1.00
***TOTAL VEC (GAL.)***	4.41	3.34	10.21	-
**TRIP AVERAGE PERFORMANCE**				
***# OF TRIPS***	31	18	94	165
***AVG. VMT (MILE)***	3.55	4.83	2.37	0.79
***AVG. VTT (SEC.)***	646	850	319	1058
***AVG. VDF***	1.21	1.55	1.00	1.00
***AVG. PWT (SEC.)***	324	294	0	-
***AVG. WKT (SEC.)***	0	990	0	-
***AVG. VEC (GAL.)***	0.14	0.19	0.11	-

*: WAK mode doesn’t have “# OF VEHICLES,” and the number of “ # OF VEHICLES” indicates the number of trips of walk mode. Similarly, all metrics titled with “vehicles,” such as “TOTAL VMT (MILE), TOTAL VTT (SEC.),” refer to pedestrian corresponding metrics. So, for example, the “TOTAL VMT (MILE)” of WAK mode represents the total pedestrian mile traveled in miles.
